# Supplementary material for: Predicting glycan structure from tandem mass spectrometry via deep learning
Source: Nat Methods. 2024 Jul 1;21(7):1206–15. doi: 10.1038/s41592-024-02314-6 (PMC11239490; doi:10.1038/s41592-024-02314-6)
Supplement: Supplementary file 1 — Supplementary Figs. 1–22. [file 41592_2024_2314_MOESM1_ESM.pdf]

nature methods

Supplementary information https://doi.org/10.1038/s41592-024-02314-6

Predicting glycan structure from tandem mass spectrometry via deep learning

In the format provided by the authors and unedited

Table of Contents

Supplementary Figure 1. Most bins in the current binning strategy contain unique fragments.

Supplementary Figure 2. Options for running CandyCrunch via *wrap_inference*.

Supplementary Figure 3. Robustness of the CandyCrunch workflow to retention time overlaps.

Supplementary Figure 4. Relative abundances obtained by CandyCrunch correlate with relative abundances from experts.

Supplementary Figure 5. Dependence of predictive performance on number of example spectra and size for each glycan.

Supplementary Figure 6. CandyCrunch learns from fragmentation of structurally similar glycans.

Supplementary Figure 7. Using biosynthetic networks for interpolative zero-shot predictions.

Supplementary Figure 8. Supplementary zero-shot prediction workflows perform robustly.

Supplementary Figure 9. A new multi-file prediction approach harmonizes retention time variation across replicates.

Supplementary Figure 10. Feature comparison between models predicting glycan structure and annotating fragments.

Supplementary Figure 11. Comparing CandyCrunch with Glycoforest.

Supplementary Figure 12. Extending expert annotation with CandyCrunch.

Supplementary Figure 13. Estimating speed of CandyCrunch and CandyCrumbs.

Supplementary Figure 14. Predicting unexpected glycans.

Supplementary Figure 15. Decision scheme for choosing the best fragment alternative within CandyCrumbs.

Supplementary Figure 16. Loss of distinctive fragmentation with decreasing spectrum quality.

Supplementary Figure 17. Signal strength of diagnostic ions across spectrum quality.

Supplementary Figure 18. Systematic fragmentation differences in GalNAcα1-3 or GlcNAcβ1-3 containing *O*-glycans.

Supplementary Figure 19. Increasing annotation comprehensiveness via CandyCrunch and CandyCrumbs.

Supplementary Figure 20. Predicting *N*-glycan structures from bluefin tuna.

Supplementary Figure 21. Analysis of inferred intermediates in biosynthetic networks of *O*-glycomes.

Supplementary Figure 22. Taxonomic distribution of our dataset.

Supplementary Table 1. Distribution of our dataset across different mass spectrometry parameters.

Supplementary Table 2. Evaluating CandyCrunch performance on different types of glycomics data.

Supplementary Table 3. Evaluating impact of training using different input data types on CandyCrunch performance.

Supplementary Table 4. Probing the effect of finer binning of fragmentation spectra on CandyCrunch performance.

Supplementary Table 5. Evaluating CandyCrunch performance on various glycan derivatizations.

Supplementary Table 6. Evaluating CandyCrunch performance on different detector types.

Supplementary Table 7. Prediction confidence is a reliable indicator of prediction trustworthiness.

Supplementary Table 8. Evaluating CandyCrunch performance on new glycomics data.

Supplementary Table 9. *O*-glycans occurring in at least 100 samples of our entire dataset, with their sequence in IUPAC-condensed and their calculated proportion of how often they occur as inferred intermediates in biosynthetic networks (0-1).

Supplementary Table 10. Glycomics data sources for training CandyCrunch.

Supplementary Table 11. List of glycans contained in training dataset.

Supplementary Table 12. Options for keyword arguments in *wrap_inference*.

Supplementary Figures

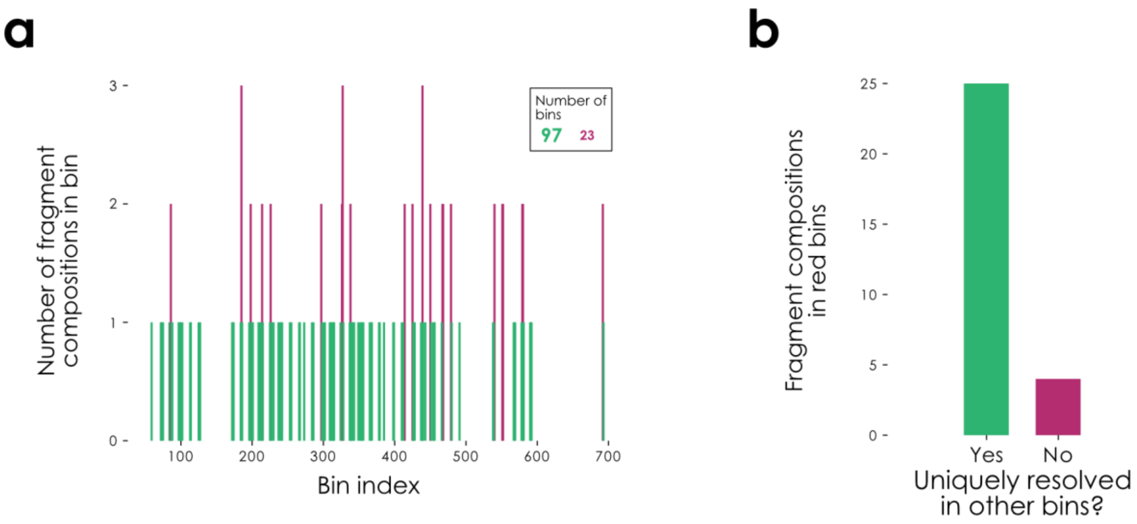

Supplementary Figure 1. Most bins in the current binning strategy contain unique fragments. a, For all precursor ions with *m/z* 1,081, we calculated the masses of all possible fragments containing at most three simple cleavages (Y, Z, B, C). The masses were then binned into 2,048 bins and unique compositions per bin were counted. We conclude that most bins only contain one fragment, for a given precursor mass. **b,** For bins with multiple fragments, we then asked for how many of those compositions a unique fragment could be found in another bin that exhibited the same composition (e.g., finding a C_1 ion for Neu5Ac to recover the same annotation information as a B_1 ion). We conclude that for nearly all cases, fragments in unique bins can be found that carry the same information, indicating that our binning strategy recovers sufficient information for annotation.

spectra_filepath	- where to load spectra from; can be either an .mzML file, an .mzXML file, or an .xlsx file if spectra have already been extracted
glycan_class	- the predominant class of glycans in sample; can be "N", "O", "lipid", or "free"
mode	- the ion mode of the mass spectrometer; either "negative" or "positive"; default is "negative"
modification	- derivatization of the glycan; can be "reduced", "permethylated", "2AA", "2AB" or "custom"; default is "reduced"
mass_tag	- if *modification* is set to "custom", the mass of the derivatization can be specified here; default is 0
lc	- type of liquid chromatography; options are "PGC", "C18", and "other"; default is "PGC"
trap	- type of mass analyzer; options are "linear", "orbitrap", "amazon", and "other"; default is "linear"
rt_min	- minimum retention time cut-off in minutes; default is 0
rt_max	- maximum retention time cut-off in minutes; default is the entire range
rt_diff	- maximum retention time difference (in minutes) to peak apex that can be grouped with that peak; default is 1.0
spectra	- whether to also output the spectra that predictions are based on; default is False
get_missing	- whether to output peaks without valid predictions but with valid compositions; default is False
mass_tolerance	- permitted variance of masses (in Da) to group peaks etc.; default is 0.5
filter_out	- composition elements to exclude; only relevant for *get_missing* or *supplement* / *experimental* below; default: {'Kdn', 'P', 'HexA', 'Pen', 'HexN', 'Me', 'PCho', 'PEtN'}
supplement	- whether to impute observed biosynthetic intermediaries from biosynthetic networks; default: True
experimental	- whether to impute missing predictions via database searches etc.; default: True
taxonomy_class	- restricting database searches in *experimental* to glycans from a taxonomic class; default: Mammalia
plot_glycans	- whether to plot glycans as SNFG images into an Excel output table; default: False

Supplementary Figure 2. Options for running CandyCrunch via *wrap_inference*. For all options where user (not developer) modification may seem reasonable, we have added descriptions and possible values for each option. Mandatory information is shown in orange, optional adjustments in blue. We note that default values reported here pertain to the state of *wrap_inference* within the CandyCrunch package and may differ from the function call in the provided Jupyter notebook.

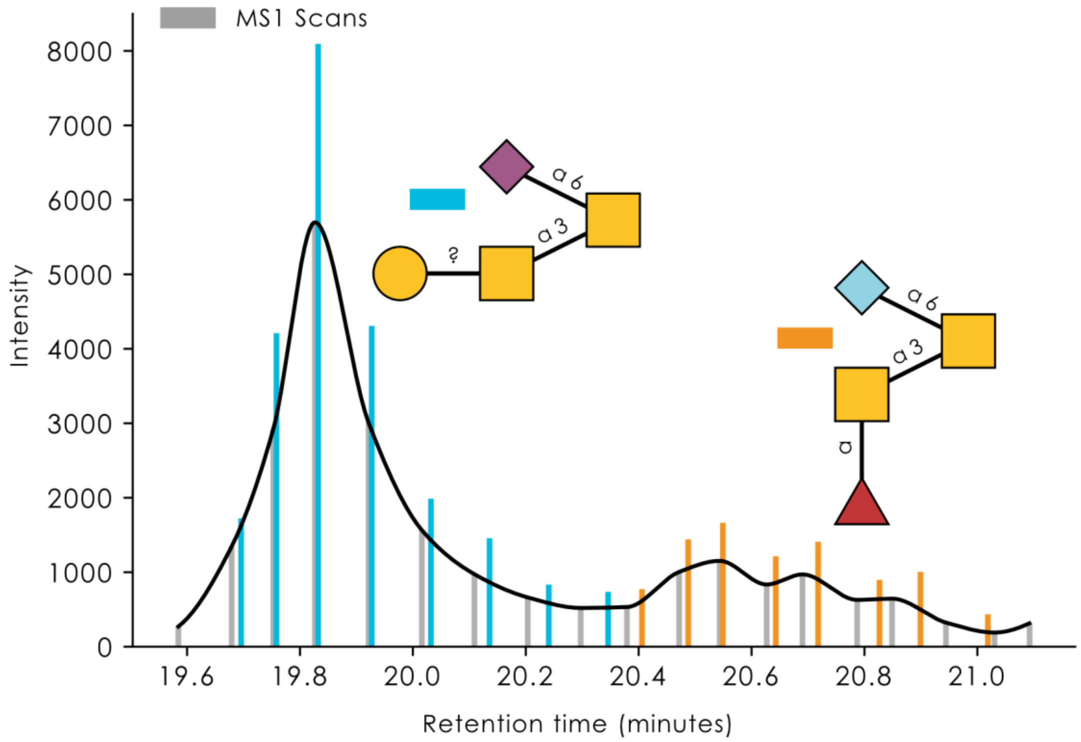

Supplementary Figure 3. Robustness of the CandyCrunch workflow to retention time overlaps. As a representative example, we analyzed the expert-annotated isomers Gal?1-?GalNAcα1-3(Neu5Acα2-6)GalNAc (*m/z* 878.54) and Fucα1-?GalNAcα1-3(Neu5Gcα2-6)GalNAc (*m/z* 878.58) in the file JC_200821F1 (GPST000151; not used for training the model). Shown are the top1 predictions of CandyCrunch for every MS2 spectrum acquired in the indicated timeframe. MS1 scans are depicted in gray. This led to the inclusion of both structures in the final output file, despite overlap in retention time and an *m/z* difference below our default cut-off (0.5). We thus conclude that our method is robust against overlaps in retention time, as long as peaks do not perfectly coincide.

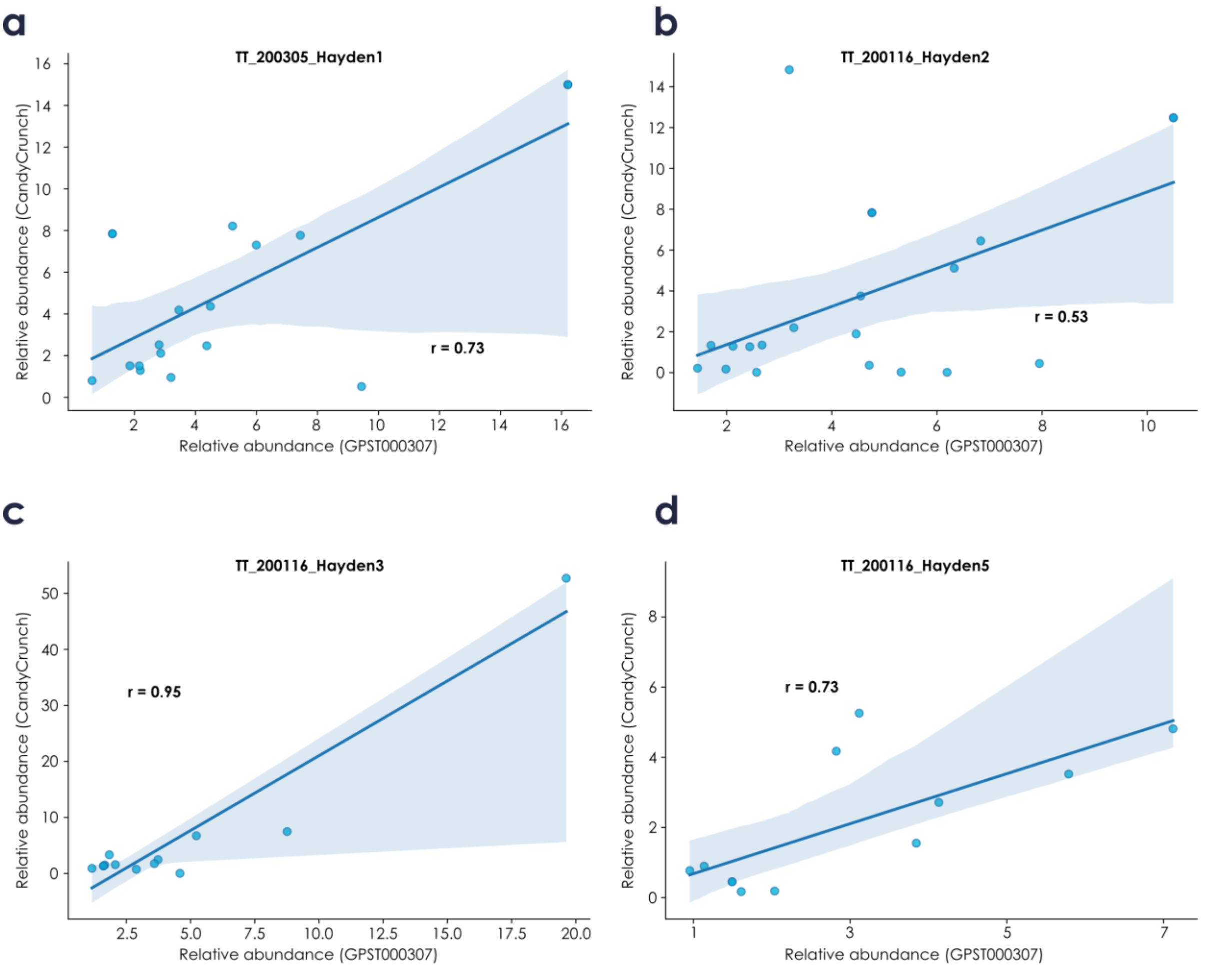

Supplementary Figure 4. Relative abundances obtained by CandyCrunch correlate with relative abundances from experts. a-d, For the four *O*-glycan raw files from GPST000307 (GlycoPOST), we extracted the ion intensity from each precursor selected for fragmentation. Then, we used CandyCrunch to predict glycan structures and paired those with relative abundances (in percent) estimated from the ion intensities. In each sample, for those glycans for which we could unequivocally establish equality between expert annotation and prediction, we compared their relative abundances quantified by the analyst as well as CandyCrunch. All correlations between experimental data and predictions were done via fitting a linear regression and r represents Pearson's correlation coefficient.

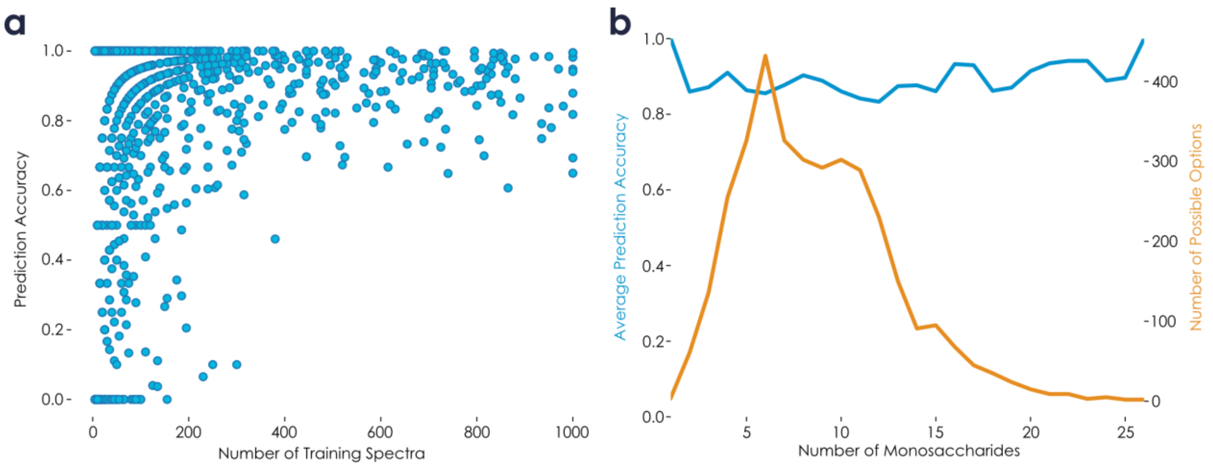

Supplementary Figure 5. Dependence of predictive performance on number of example spectra and size for each glycan. a, For all 1,497 unique glycans occurring in the independent test set, the prediction performance of CandyCrunch on that glycan in the independent test set is plotted against the number of example spectra of that glycan in the training set. The plot shows that CandyCrunch exhibits high predictive performance for most glycans and that relatively few example spectra for a new glycan are needed to achieve high predictive performance. **b,** Similarly, we plotted the average prediction per glycan size (i.e., number of monosaccharides) in the independent test set. While this shows a stable prediction accuracy across glycan sizes, we note that the number of possible options (i.e., how many glycans of size 26 are in our dataset) drastically decreases with glycan size, suggesting that the indicated accuracy for very large glycans might be overly optimistic.

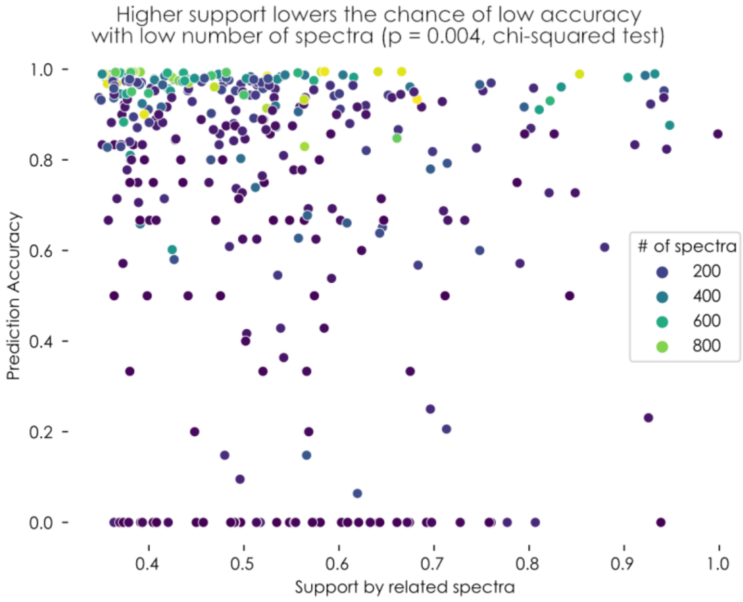

Supplementary Figure 6. CandyCrunch learns from fragmentation of structurally similar glycans. For all glycans in our independent validation set with fewer than 1,000 spectra in the training dataset, we catalogued their prediction accuracy. Then, we calculated a support score, to indicate how well their structural features are represented in other spectra of the training dataset. For this, we used the cosine similarity of the motif vectors of the target glycan and another glycan (also used in our structure distance loss, see Methods) as a weighting factor to multiply the number of training spectra, summed up across all other glycans. This score was then normalized to a range between zero and one, by dividing by the maximum support score. We then tested the hypothesis that, in cases of few training spectra, higher values of support lowered the chances of obtaining low accuracy for a structure, bolstering our claim of cross-training on structurally related glycans. For this, we used a support threshold of 0.5 and a prediction accuracy of 0.5 within a two-tailed chi-squared test, resulting in the conclusion that higher support values indeed may low-accuracy predictions less likely (p = 0.004).

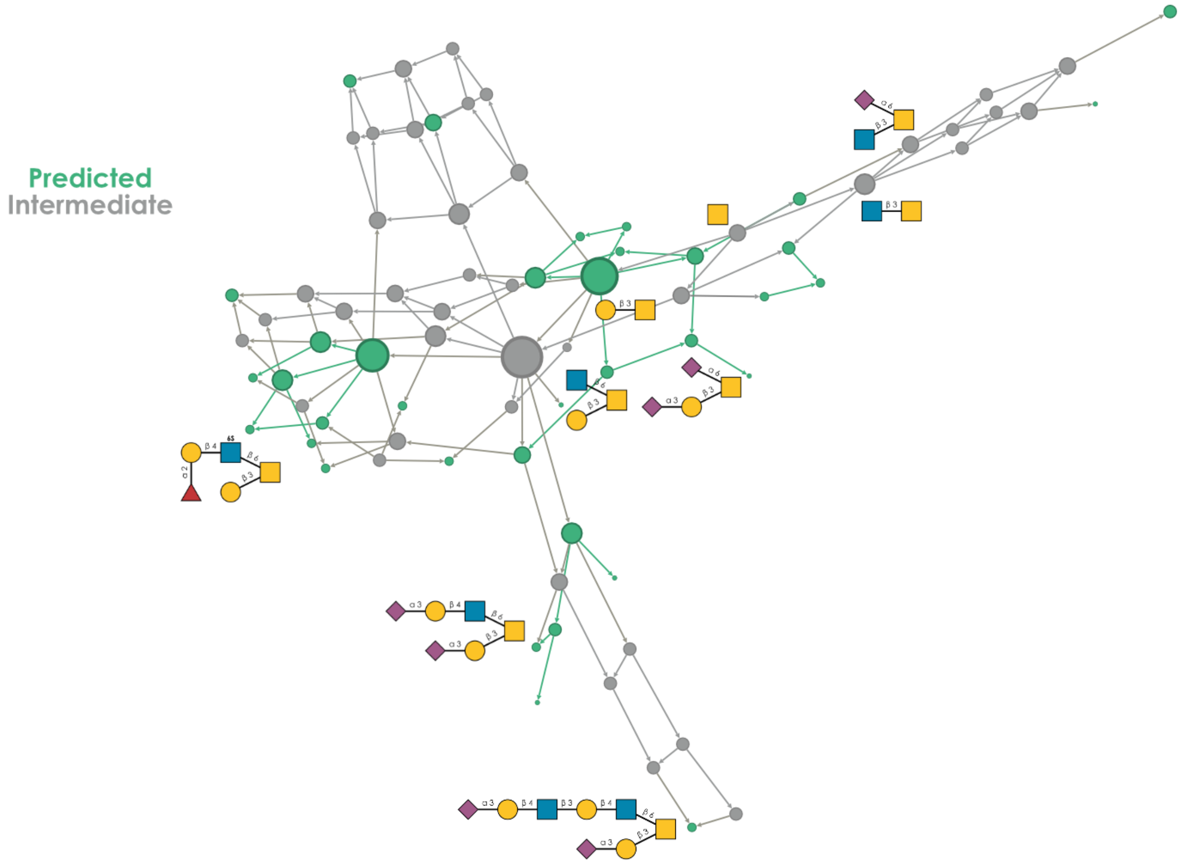

Supplementary Figure 7. Using biosynthetic networks for interpolative zero-shot predictions. The biosynthetic network was constructed as previously described[1], from glycan predictions of the example file KM-O-gly-CaCo-2-900sps-3l-3psi-280-meOH-5D_3_1-0006-0006.mzML from GPST000256. CandyCrunch predictions are colored in green, and inferred intermediates connecting these predictions are colored in gray. Nodes are scaled by degree. Selected structures are drawn via their SNFG representation.

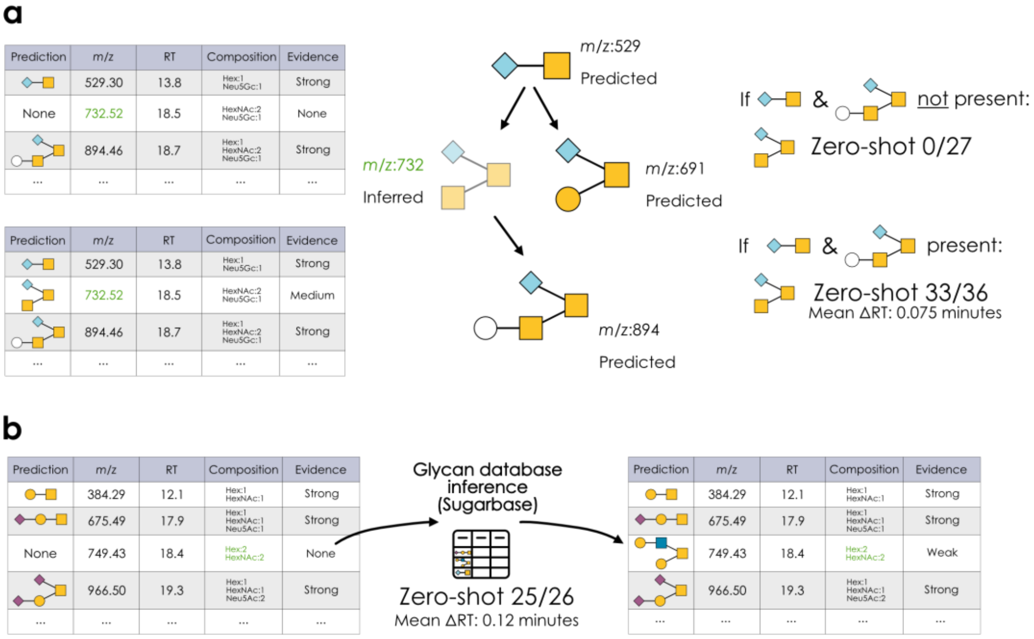

Supplementary Figure 8. Supplementary zero-shot prediction workflows perform robustly. a, Biosynthetic modeling to recover zero-shot intermediates. To test this workflow, we removed the structure GalNAcα1-3(Neu5Gcα2-6)GalNAc from the prediction library, converting this prediction task into a zero-shot case. Then, we analyzed 69 glycomics files from GPST000151 (GlycoPOST), to investigate whether biosynthetic modeling (*supplement* = *True* in our CandyCrunch package) can recover this structure, compared to expert annotation. We confirm that this works robustly, including a near-perfect match to the annotated retention time, as long as the assumptions for biosynthetic modeling (the presence of any precursor and any successor in the file, though not necessarily the immediate neighbors) are met. In this case, the retention time match is achieved by the fact that the only eligible peak (no valid prediction and containing diagnostic fragments etc.) was the correct peak. **b,** Database inference approaches are another effective zero-shot means. If neither prediction nor biosynthetic modeling led to a successful candidate, we employ database inference (*experimental* = *True*), using the glycowork-internal database to search for glycans with matching compositions. We restrict these searches to glycans of the relevant class, a composition element blacklist (excluding specified components), as well as a specified taxonomic class (default: Mammalia). Still, we caution that this approach might retrieve several matching sequences. For the example of Galβ1-4GlcNAcβ1-6(Galβ1-3)GalNAc, we removed it from our prediction library and assessed how well the database inference approach recovered it. Probing 69 glycomics files from GPST000151, we report an average success rate of >96%, confirming the usefulness of this method.

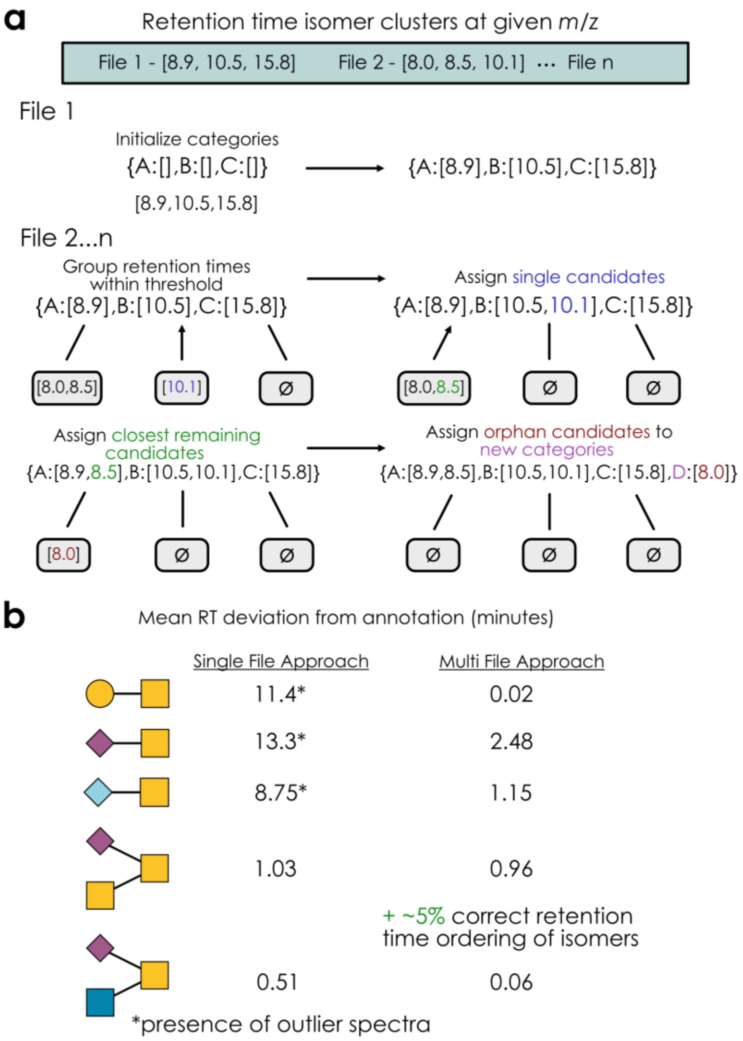

Supplementary Figure 9. A new multi-file prediction approach harmonizes retention time variation across replicates. a, Schematic view of the harmonization approach using the new *wrap_inference_batch* function, in which the goal is to align isomers across files, in the presence of varying retention times. **b,** Comparing retention time deviations of predictions and expert annotations between *wrap_inference* (single file) and *wrap_inference_batch* (multi file) using 69 glycomics files from GPST000151. In general, the multi-file approach led to an improved annotation of the correct retention time in each file, for a given glycan. In some cases, this even resulted in a correction of the retention time ordering of isomers (e.g., *m/z* 716), in comparison to the single-file approach. We note that this assessment is sensitive to single outlier spectra at high retention times (e.g., *m/z* 384 close to 40 minutes in a 45-minute LC run) that can create high averaged retention time deviations in the single-file approach, which is alleviated in the multi-file approach. In cases where this causes issues for the single-file approach, we recommend using the "rt_max" keyword argument in *wrap_inference* or setting "experimental = True", for the removal of retention time outliers.

a

	Glycoforest (2017)	GlycoDeNovo2 (2022)	CandyCrunch (ours)
**Approach**	Consensus spectrum network search	Composition constrained topology search	Dilated residual neural network classification
**Language**	Java	MATLAB	Python
**Runtime**	Minutes	n/a	Seconds
**Input**	mzXML	List of peaks	mzML/mzXML
**Web hosted**	Yes	No	Yes
**Glycan types**	*O*-glycans	*N*-, *O*-, lipid, free glycans	*N*-, *O*-, lipid, free glycans
**Stereochemical resolution**	No	Yes	Yes
**Linkage type resolution**	No	No	Yes
**Supported post-biosynthetic modifications**	Sulfation	-	Sulfation, Phosphorylation, Acetylation, etc.
**Experimental setup generalizability**	-	-	Ion mode, liquid chromatography, glycan modification, etc.

b

	GlycoWorkbench (2008)	glypy (2019)	CandyCrumbs (ours)
**Language**	Java	Python	Python
**SNFG Output**	Yes	No	Yes
**IUPAC Output**	No	No	Yes
**Cross-ring fragments**	Yes	No	Yes
**Cross-ring validity checks**	No	No	Yes
**Linkage type resolution**	No	No	Yes
**Optional fragmentation types**	Yes	No	No
**Programmatic integration**	No	Yes	Yes
**Fragment prioritization**	No	No	Yes

Supplementary Figure 10. Feature comparison between models predicting glycan structure and annotating fragments. a-b, We compared current state-of-the-art methods for the tasks of predicting glycan structure from MS/MS data (a) and annotating therein resulting fragment ions (b) to assess the capabilities of our CandyCrunch and CandyCrumbs methods, respectively. Representative features were chosen to compare the current strengths and weaknesses of each method as best as possible. Green coloring indicates feature values that were judged to be more desirable. We conclude that, based on these metrics, CandyCrunch and CandyCrumbs present the best-in-class solution to the respective problem.

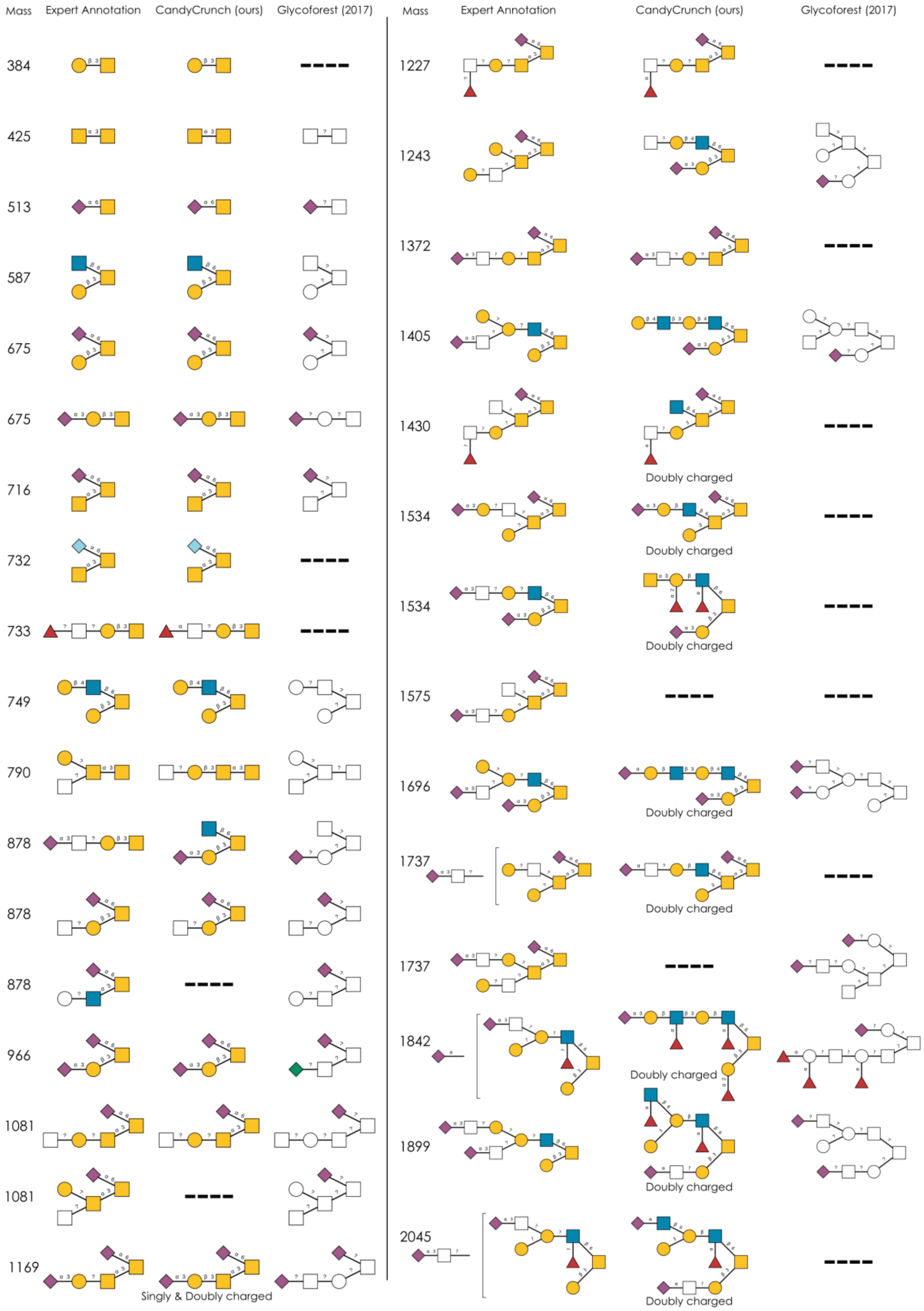

Supplementary Figure 11. Comparing CandyCrunch with Glycoforest. For JC_131210PMpx5.mzML, not used for training CandyCrunch but used for developing Glycoforest, we compared predictions by both methods for all expert-annotated structures. Additional CandyCrunch predictions exist beyond expert annotations. Deviations from expert annotations (e.g., 878-1) are not necessarily errors. Glycans are shown via the SNFG, with isomers ordered by retention time.

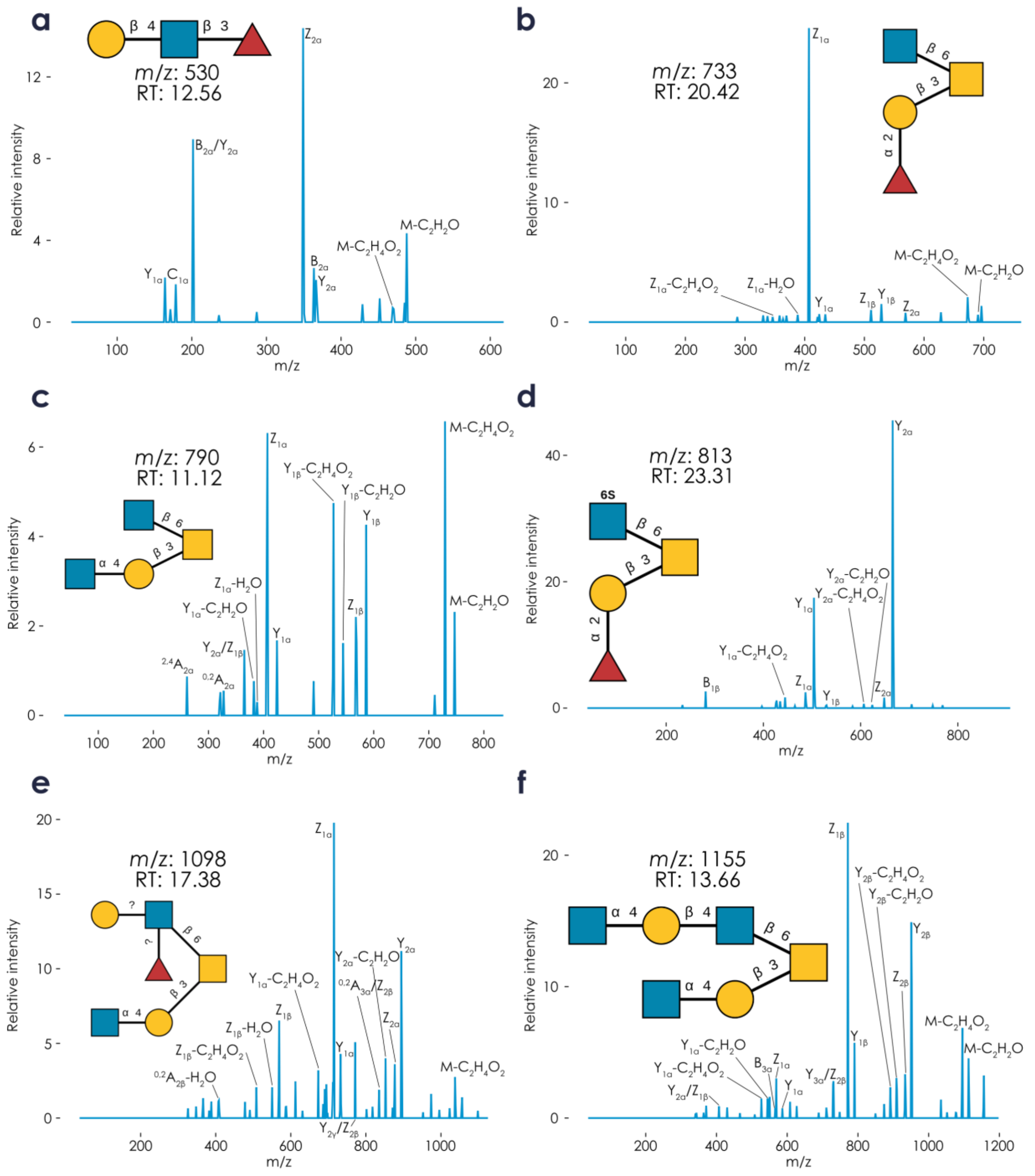

Supplementary Figure 12. Extending expert annotation with CandyCrunch. a-f, We predicted all *O*-linked glycan structures from the raw MS/MS file JC_171002Y1 (from previous work[2]). Starting from the smallest structures, we chose the first six predicted structures that were not contained in the expert annotation (either new masses or additional isomers at different retention times). We assume that most of these structures, except for (a), based on their abundance and biosynthetic character, could stem from the remnants of the porcine gastric mucin sample used to calibrate the MS instrument, showcasing the exceptional sensitivity of CandyCrunch. The associated MS/MS spectra used for prediction, together with their *m/z* values, retention times, and predicted structures are shown with their annotated fragments in the Domon-Costello nomenclature.

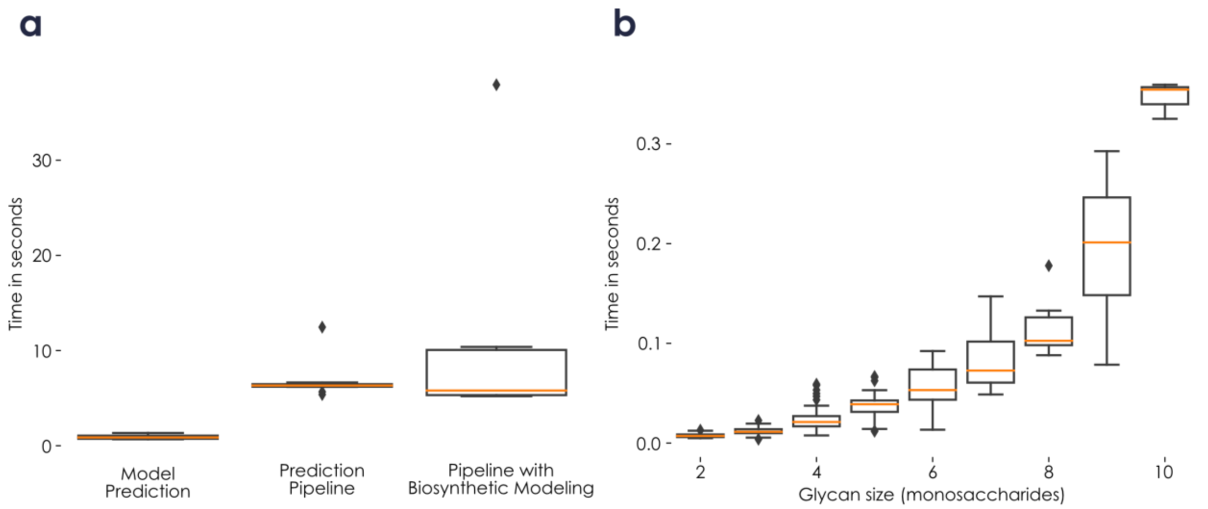

Supplementary Figure 13. Estimating speed of CandyCrunch and CandyCrumbs. a, Using all raw MS/MS files from previous work[2] (n = 10), we extracted all spectra with *process_mzML_stack()* and recorded the time in seconds for (i) predicting all spectra with CandyCrunch, (ii) predicting and curating all spectra, and (iii) predicting and curating, including biosynthetic modeling for zero-shot predictions. Predictions were performed using a single Nvidia A100 GPU and the results are depicted via boxplots. **b,** On the same files as in (a), we used all 205 top1 glycan predictions (n = 205) and their spectra to annotate their fragments via CandyCrumbs. Computation time per glycan is plotted against glycan size, using an Intel® Xeon® CPU @ 2.00GHz. Variance within one size can be explained by different degrees of branching. For both (a) and (b), box plots used the median as the center line and the 25th (Q1) and 75th (Q3) percentile as the lower and upper edge of the box. The whiskers extend to the first data point within Q1 - 1.5 * IQR (interquartile range) and to the last datapoint within Q3 + 1.5 * IQR and outlier values outside this range are depicted as diamonds.

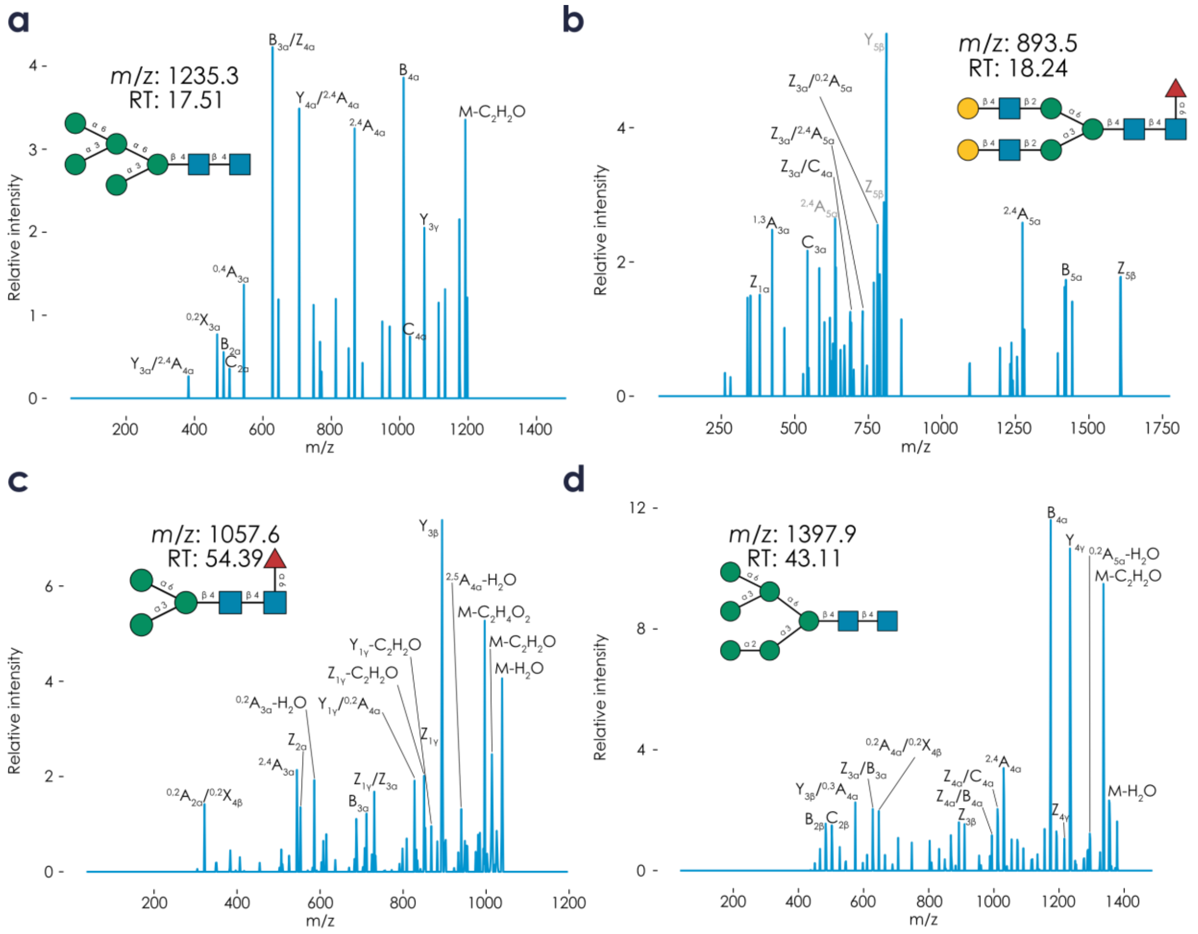

Supplementary Figure 14. Predicting unexpected glycans. a-d, Shown are example CandyCrunch predictions of co-released *N*-linked glycans in preparations of *O*-linked glycans that were not annotated in the respective publications. The examples are from JC_171002Y2.mzML (a-b; from previous work[2]) and KM-O-gly-CaCo-2-900sps-3l-3psi-280-meOH-5D_3_1-0006-0006.mzML (c-d; GPST000256). The associated MS/MS spectra used for prediction, together with their *m/z* values, retention times, and predicted structures are shown with their fragments, annotated by CandyCrumbs, in the Domon-Costello nomenclature. Doubly-charged fragment ions are denoted with gray text.

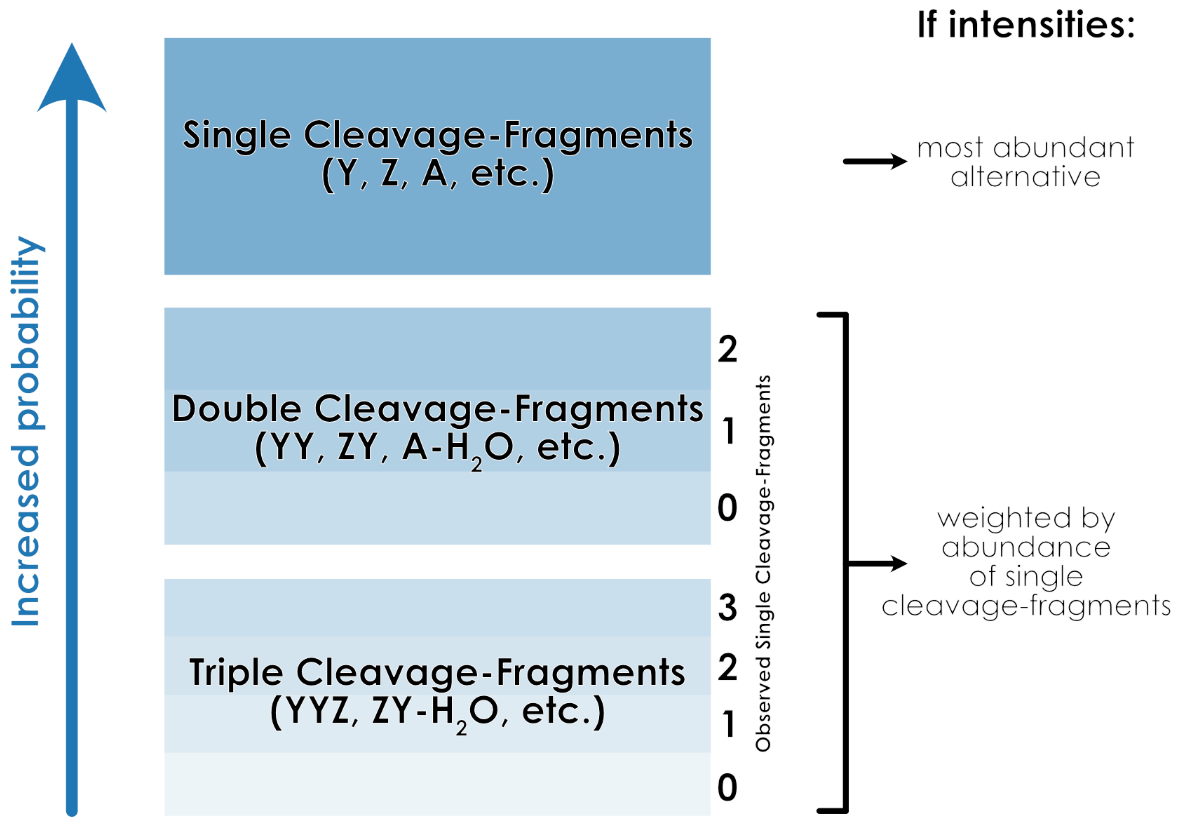

Supplementary Figure 15. Decision scheme for choosing the best fragment alternative within CandyCrumbs. From the fragment possibilities, up to triple cleavage-fragments, that explained a given *m/z* within a specified variance, we selected the most probable explanation via heuristics and domain knowledge. We note that this behavior only applies when *simplify* = *True* when using CandyCrumbs, otherwise all possible fragments will be returned. For *simplify* = *True*, less complex fragmentation (e.g., single cleavage-fragments) were always preferred over more elaborate fragmentation patterns. Further, evidence of constituent single cleavage-fragment ions (e.g., Y) in a spectrum was interpreted as evidence for the double cleavage-fragment possibilities comprised of these single cleavage-fragment ions (e.g., YZ). We recognize this as a probabilistic heuristic, which may not always hold true. If *m/z* intensities were also supplied, this type of evidence was weighted by the observed abundance of the respective single cleavage-fragments (e.g., Y*intensity(Y)), with the assumption that more prominent single cleavage-fragment ions were also more likely to spawn compound fragmentation products.

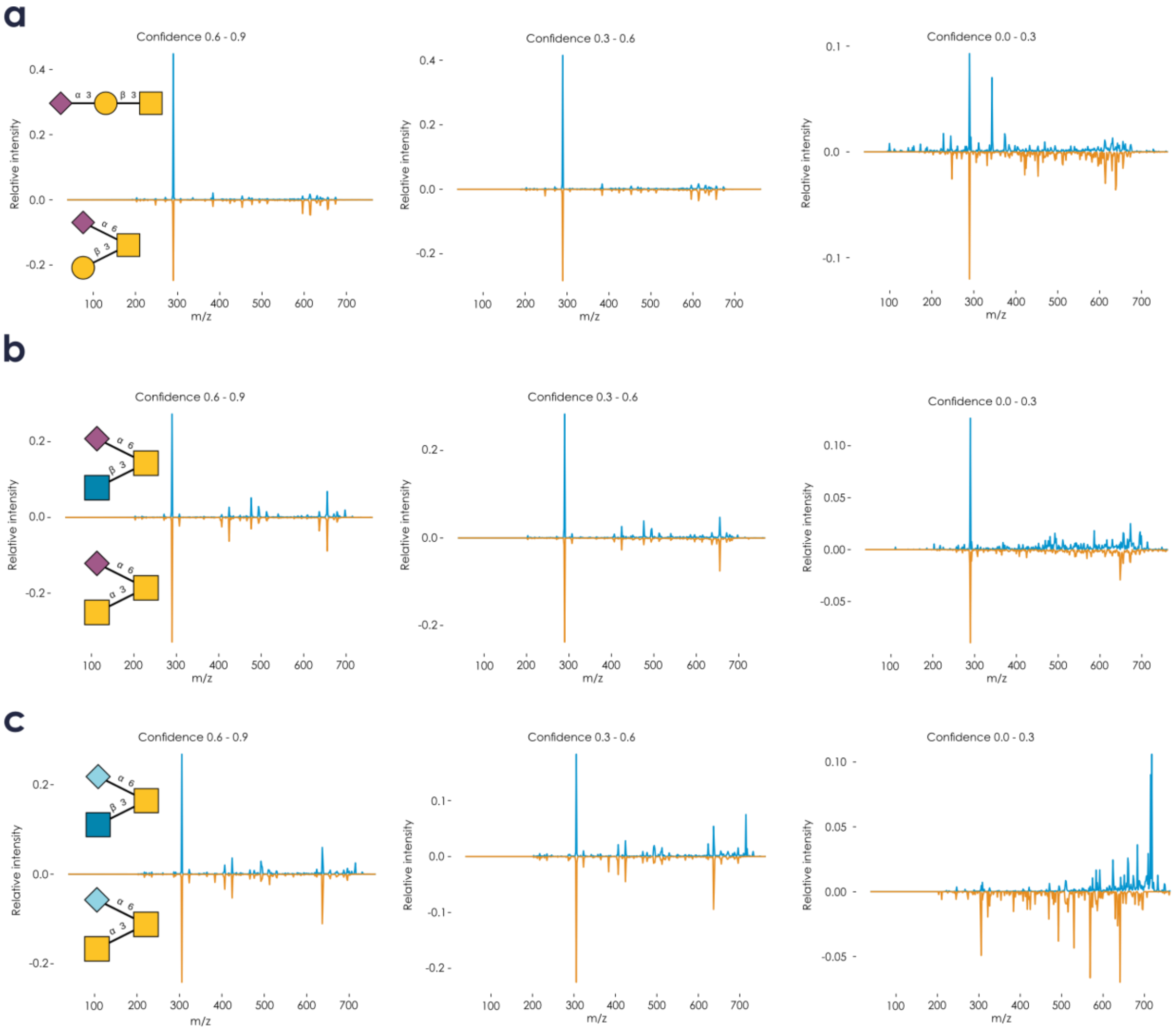

Supplementary Figure 16. Loss of distinctive fragmentation with decreasing spectrum quality. a-c, Similar to Figure 2, averaged spectra of Neu5Acα2-3Galβ1-3GalNAc / Galβ1-3(Neu5Acα2-6)GalNAc (a), GlcNAcβ1-3(Neu5Acα2-6)GalNAc / GalNAcα1-3(Neu5Acα2-6)GalNAc (b), and GlcNAcβ1-3(Neu5Gcα2-6)GalNAc / GalNAcα1-3(Neu5Gcα2-6)GalNAc (c) are juxtaposed for several bins of prediction confidence.

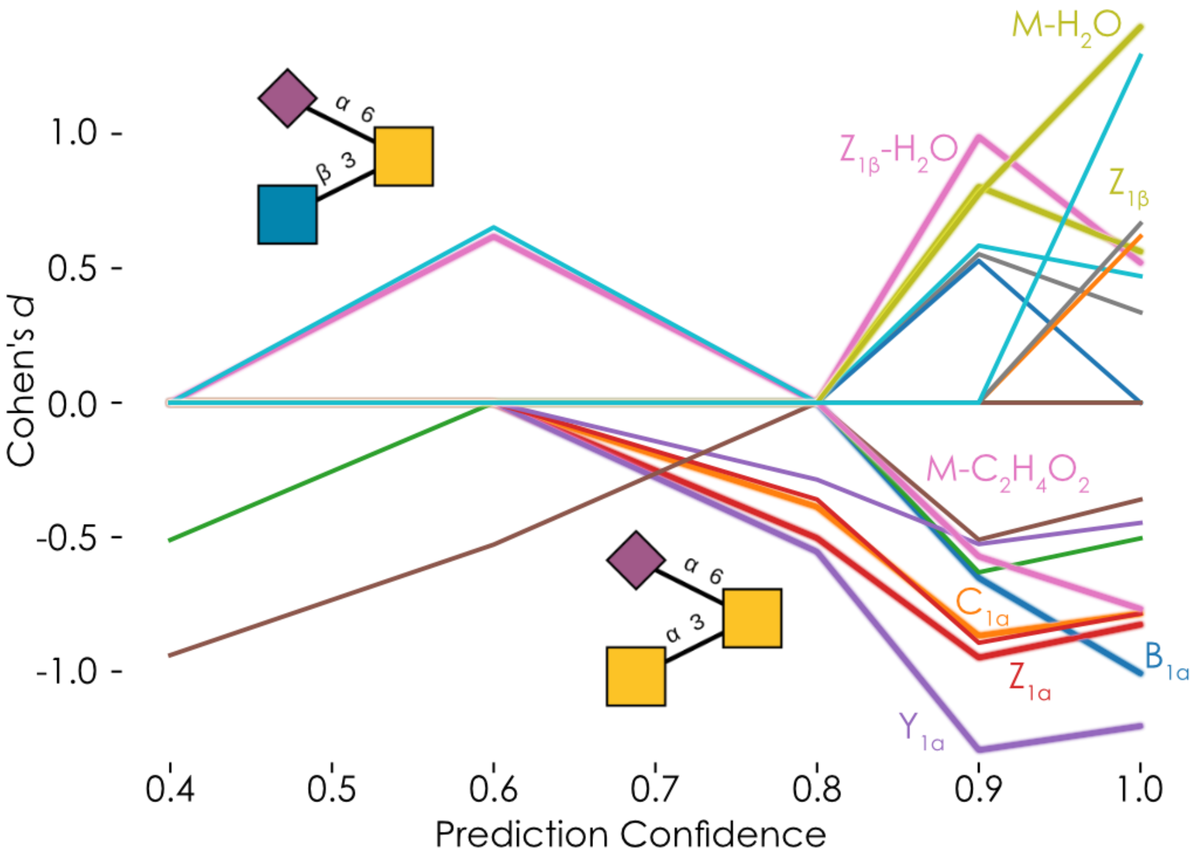

Supplementary Figure 17. Signal strength of diagnostic ions across spectrum quality. For the comparison of GlcNAcβ1-3(Neu5Acα2-6)GalNAc / GalNAcα1-3(Neu5Acα2-6)GalNAc, we binned spectra according to prediction confidence, as a measure of spectrum quality, and calculated the effect sizes of significantly different peaks in each bin. Significance was established via two-tailed Welch's t-tests of the normalized peak intensities ($p < 0.05$), with a Holm-Šídák correction for multiple testing. Effect size was calculated as Cohen's *d*. Positive effect size means more prevalent in the first structure. Line colors distinguish the different fragments. Example fragments, annotated via CandyCrumbs, are noted next to their line plots in Domon-Costello nomenclature. Exact p-values are: M-H_2O = 0.000170, $Z_{1\beta}$-H_2O = 0.000609, $Z_{1\beta}$ = 0.001496, M-$C_2H_4O_2$ = 0.002027, $C_{1\alpha}$ = 0.002717, $Z_{1\alpha}$ = 0.001621, $B_{1\alpha}$ = 0.000812, $Y_{1\alpha}$ = 0.000678.

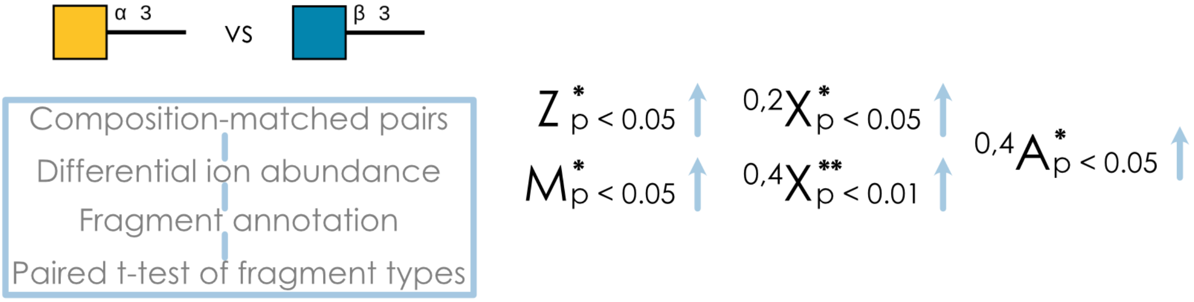

Supplementary Figure 18. Systematic fragmentation differences in GalNAcα1-3 or GlcNAcβ1-3 containing *O*-glycans. All *O*-glycan spectra containing GalNAcα1-3 or GlcNAcβ1-3 with confidence between 0.7 and 1.0 were used in the described workflow to ascertain their characteristic types of fragmentation. p-values stem from two-tailed Welch's t-tests, corrected with a Holm-Šídák correction for multiple testing. Exact p-values are: Z = 0.017176, M = 0.040757, 02X = 0.019407, 04X = 0.003748, 04A = 0.025705.

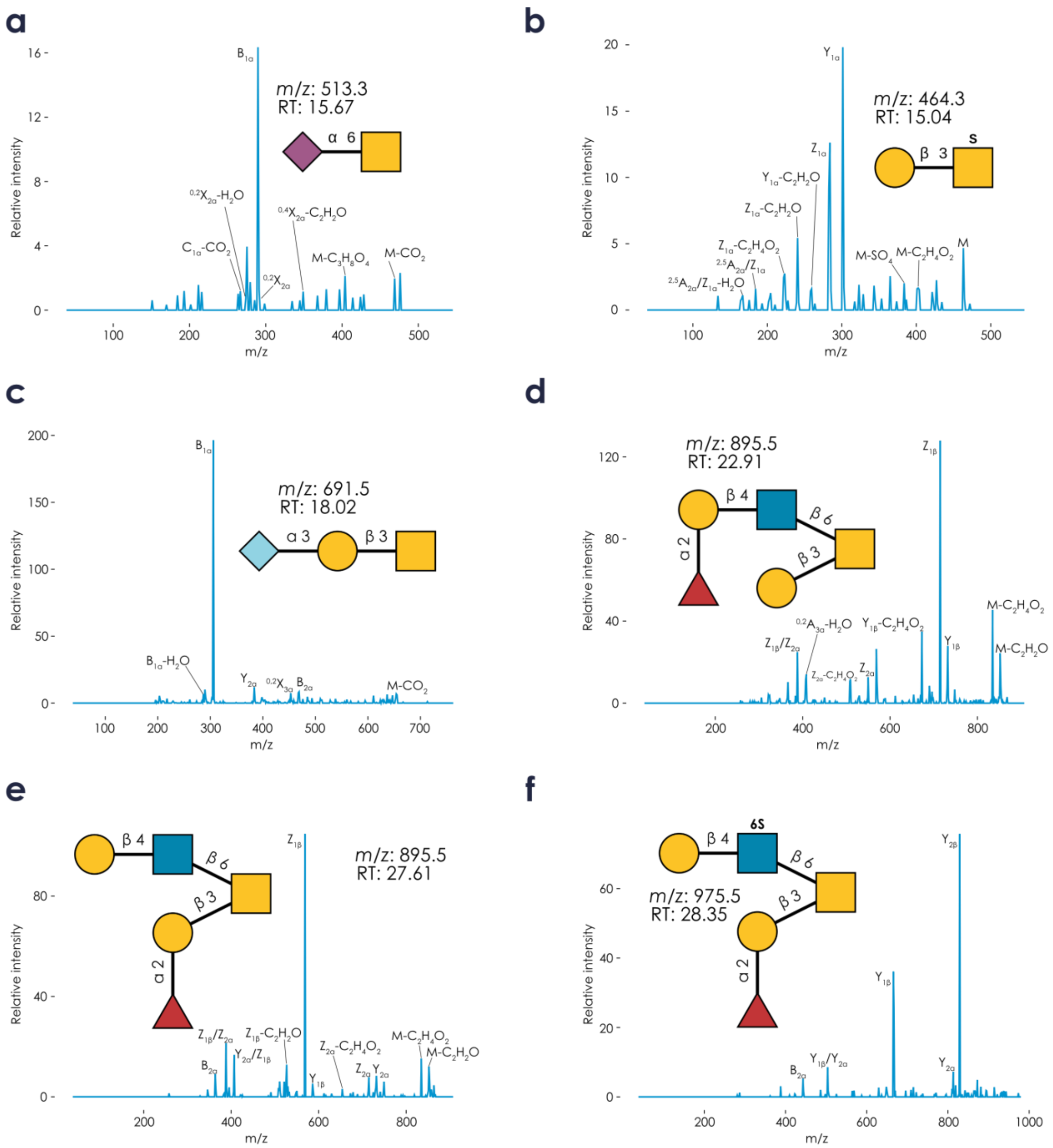

Supplementary Figure 19. Increasing annotation comprehensiveness via CandyCrunch and CandyCrumbs. a-f, We used CandyCrunch to predict all glycan structures from the file TT_200116Hayden_5 in GPST000307, containing murine intestinal *O*-glycans. We then chose example predictions which were absent from the associated annotation on GPST000307. The associated MS/MS spectra used for prediction, together with their *m/z* values, retention times, and predicted structures are shown with their fragments, annotated by CandyCrumbs, in the Domon-Costello nomenclature.

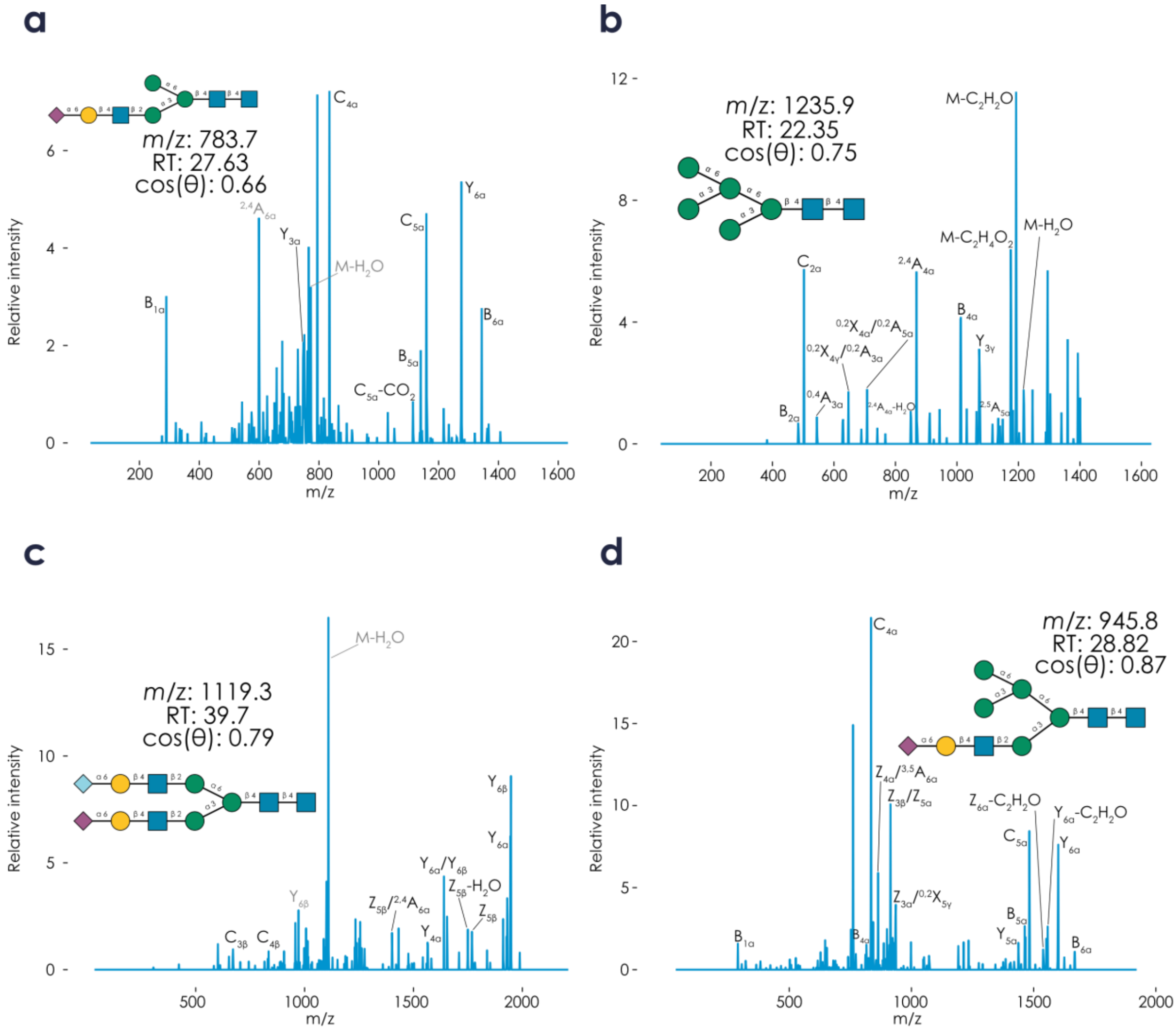

Supplementary Figure 20. Predicting *N*-glycan structures from bluefin tuna. a-d, Using the raw file LC_SBT_blood_NG_TR1_22082019_BB3_01_2514.d.mzXML from GPST000182, measuring reduced *N*-glycans from southern bluefin tuna (*Thunnus maccoyii*) blood in negative ion mode, we used our CandyCrunch pipeline to predict and curate *N*-glycan structures. Example predictions are shown with their *m/z* value and retention time in minutes, together with their MS^2 spectrum, with abundant fragments annotated in Domon-Costello nomenclature. Doubly-charged fragment ions are denoted with gray text. Also shown is the cosine similarity, cos(θ), of the shown spectrum and the averaged spectrum of all negative ion mode spectra of reduced glycans of the predicted structure with a confidence above 0.5 (see Fig. 2 for background).

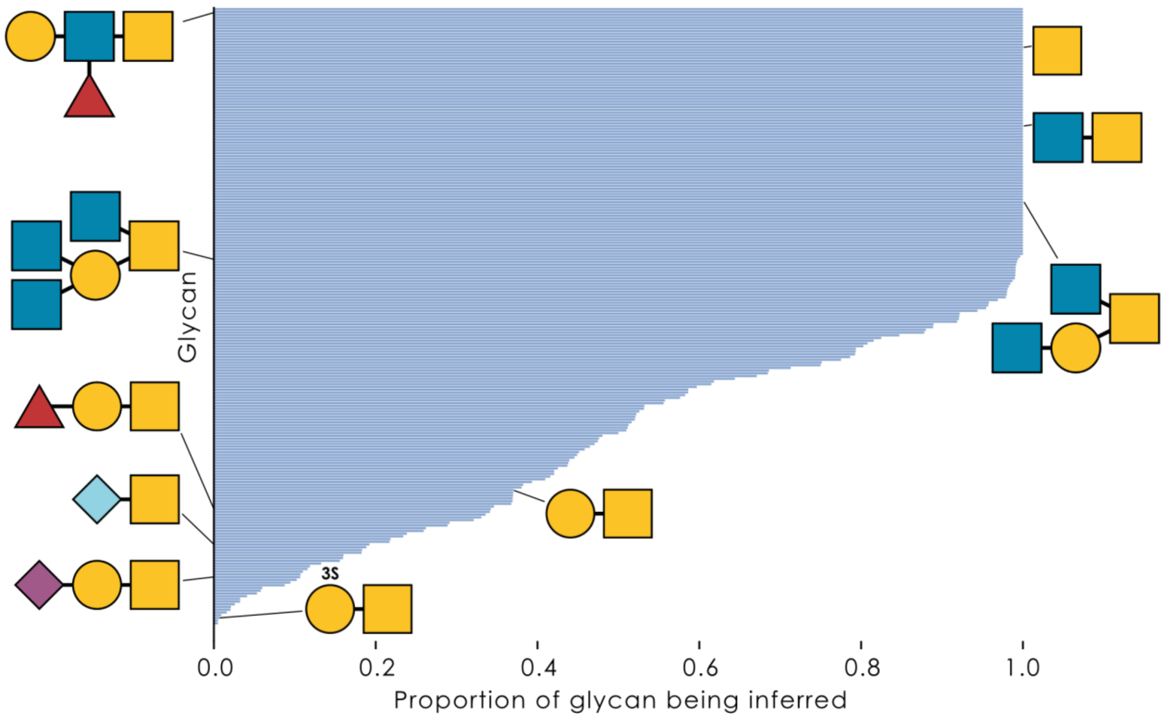

Supplementary Figure 21. Analysis of inferred intermediates in biosynthetic networks of *O*-glycomes. For all 1,003 biosynthetic networks we calculated from our *O*-glycome samples, we quantified whether a glycan was being present as an observed structure or an inferred intermediate to complete the network. We then calculated the ratio of these two quantities as an indicator of how likely it was to observe a given glycan via LC-MS/MS. Shown are the glycans that were present in the networks of at least 100 samples in our dataset, with example structures annotated via the SNFG.

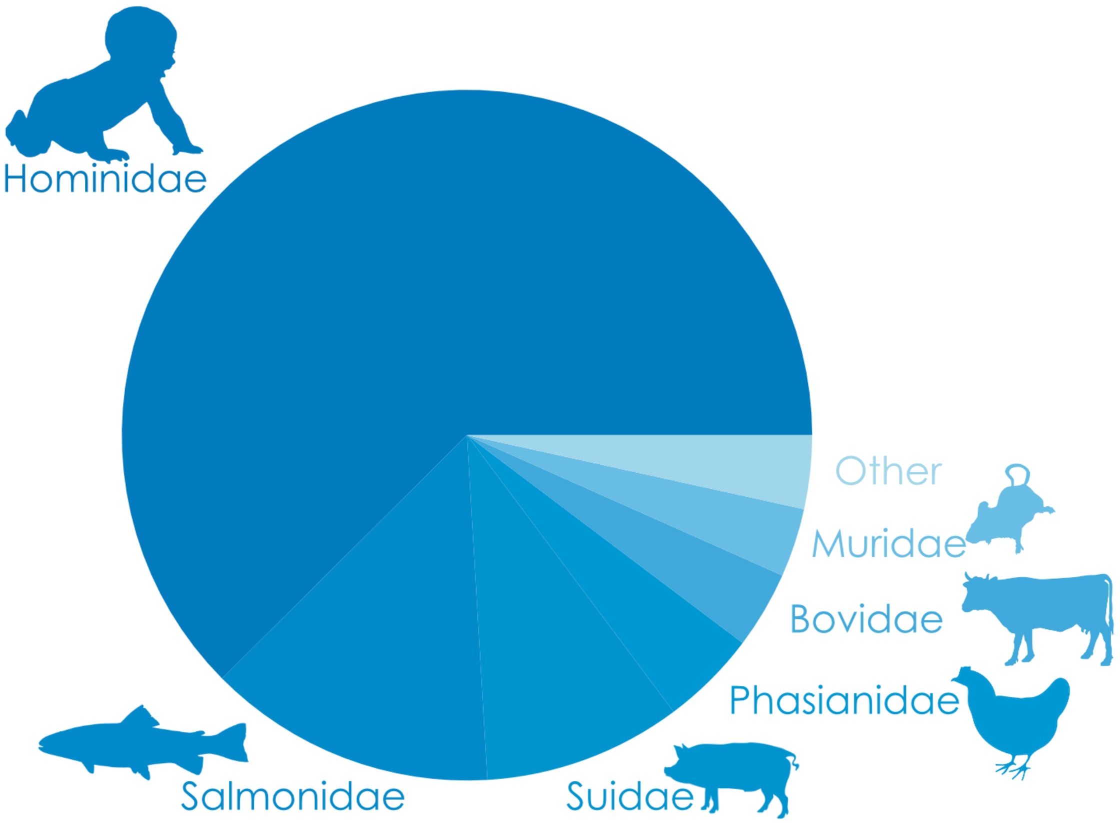

Supplementary Figure 22. Taxonomic distribution of our dataset. For our entire dataset of ~500,000 annotated mass spectra, we labeled each spectrum with the species it stemmed from and catalogued this information at the taxonomic family level, displaying relative proportions for the entire dataset. Taxonomic groups that are only distantly related to the displayed groups are likely to yield poorer predictions, due to stronger deviations from glycans seen during training. Animal illustrations were taken from https://www.phylopic.org/.

Supplemental References

1. Thomès, L., Karlsson, V., Lundstrøm, J. & Bojar, D. Mammalian milk glycomes: Connecting the dots between evolutionary conservation and biosynthetic pathways. *Cell Reports* **42**, 112710 (2023).
2. Kouka, T. *et al*. Computational Modeling of O-Linked Glycan Biosynthesis in CHO Cells. *Molecules* **27**, 1766 (2022).
