# Supplementary material for: Predicting glycan structure from tandem mass spectrometry via deep learning
Source: Nat Methods. 2024 Jul 1;21(7):1206–15. doi: 10.1038/s41592-024-02314-6 (PMC11239490; doi:10.1038/s41592-024-02314-6)
Supplement: Supplementary file 2 — Reporting Summary [file 41592_2024_2314_MOESM2_ESM.pdf]

nature portfolio

Corresponding author(s): Daniel Bojar

Last updated by author(s): Apr 2, 2024

Reporting Summary

Nature Portfolio wishes to improve the reproducibility of the work that we publish. This form provides structure for consistency and transparency in reporting. For further information on Nature Portfolio policies, see our Editorial Policies and the Editorial Policy Checklist.

Statistics

For all statistical analyses, confirm that the following items are present in the figure legend, table legend, main text, or Methods section.

n/a	Confirmed	
☐	☒	The exact sample size (*n*) for each experimental group/condition, given as a discrete number and unit of measurement
☒	☐	A statement on whether measurements were taken from distinct samples or whether the same sample was measured repeatedly
☐	☒	The statistical test(s) used AND whether they are one- or two-sided<br>*Only common tests should be described solely by name; describe more complex techniques in the Methods section.*
☒	☐	A description of all covariates tested
☐	☒	A description of any assumptions or corrections, such as tests of normality and adjustment for multiple comparisons
☐	☒	A full description of the statistical parameters including central tendency (e.g. means) or other basic estimates (e.g. regression coefficient) AND variation (e.g. standard deviation) or associated estimates of uncertainty (e.g. confidence intervals)
☐	☒	For null hypothesis testing, the test statistic (e.g. *F*, *t*, *r*) with confidence intervals, effect sizes, degrees of freedom and *P* value noted<br>*Give P values as exact values whenever suitable.*
☒	☐	For Bayesian analysis, information on the choice of priors and Markov chain Monte Carlo settings
☒	☐	For hierarchical and complex designs, identification of the appropriate level for tests and full reporting of outcomes
☐	☒	Estimates of effect sizes (e.g. Cohen's *d*, Pearson's *r*), indicating how they were calculated

Our web collection on statistics for biologists contains articles on many of the points above.

Software and code

Policy information about availability of computer code

Data collection	Python (version 3.9), glycowork (version 1.1.0), CandyCrunch (version 0.3.0), pymzML (version 2.5.2), pyteomics (version 4.6), msconvert (version 3.0.23158-5fbe5ea)
Data analysis	Python (verson 3.9), glycowork (version 1.1.0), CandyCrunch (version 0.3.0), PyTorch (version 2.1.0), NetworkX (version 3.0), scikit-learn (version 1.2.2), statsmodels (version 0.13.5), scipy (version 1.10.1), scikit-bio (version 0.5.8), https://colab.research.google.com/github/BojarLab/CandyCrunch/blob/main/CandyCrunch.ipynb, https://github.com/BojarLab/CandyCrunch

For manuscripts utilizing custom algorithms or software that are central to the research but not yet described in published literature, software must be made available to editors and reviewers. We strongly encourage code deposition in a community repository (e.g. GitHub). See the Nature Portfolio guidelines for submitting code & software for further information.

Data

Policy information about availability of data

All manuscripts must include a data availability statement. This statement should provide the following information, where applicable:

- Accession codes, unique identifiers, or web links for publicly available datasets
- A description of any restrictions on data availability
- For clinical datasets or third party data, please ensure that the statement adheres to our policy

All relevant data, including their data provenance with accession IDs from GlycoPOST, MassIVE, UniCarb-DB, UniCarb-DR, or NIST, can be found at Zenodo under the doi:10.5281/zenodo.10029271 or is contained within the supplementary material. The 196 data sources are listed in Supplementary Table 10.

Human research participants

Policy information about studies involving human research participants and Sex and Gender in Research.

Reporting on sex and gender	n/a
Population characteristics	n/a
Recruitment	n/a
Ethics oversight	n/a

Note that full information on the approval of the study protocol must also be provided in the manuscript.

Field-specific reporting

Please select the one below that is the best fit for your research. If you are not sure, read the appropriate sections before making your selection.

[x] Life sciences [ ] Behavioural & social sciences [ ] Ecological, evolutionary & environmental sciences

For a reference copy of the document with all sections, see nature.com/documents/nr-reporting-summary-flat.pdf

Life sciences study design

All studies must disclose on these points even when the disclosure is negative.

Sample size	All publicly available LC-MS/MS glycomics data using electrospray ionization were gathered from all available sources (cut-off September 2023). This resulted in 625,547 glycan spectra.
Data exclusions	We excluded spectra which we could not unambiguously pair with an expert glycan annotation. This resulted in 489,103 spectra that were labeled with a defined structure.
Replication	All results could be successfully replicated for at least three times, for instance with the same model trained with different seeds.
Randomization	Training and validation data were split randomly on a file basis, so that no spectra of the same raw file could be found in both training and validation set, improving robustness.
Blinding	For all assessments of model performance, experimenters were blinded to the expert annotation, which was only revealed at the time of evaluation.

Reporting for specific materials, systems and methods

We require information from authors about some types of materials, experimental systems and methods used in many studies. Here, indicate whether each material, system or method listed is relevant to your study. If you are not sure if a list item applies to your research, read the appropriate section before selecting a response.

Materials & experimental systems

n/a	Involved in the study	
☒	☐	Antibodies
☒	☐	Eukaryotic cell lines
☒	☐	Palaeontology and archaeology
☒	☐	Animals and other organisms
☒	☐	Clinical data
☒	☐	Dual use research of concern

Methods

n/a	Involved in the study	
☒	☐	ChIP-seq
☒	☐	Flow cytometry
☒	☐	MRI-based neuroimaging
